# Notes on distribution of *Simulium damnosum s. l.* along Atbara River in Galabat sub-focus, eastern Sudan

**DOI:** 10.1186/s12879-019-4113-1

**Published:** 2019-05-28

**Authors:** Isam M. A. Zarroug, Arwa Elaagip, Suhaib G. Gumaa, Altayeb K. Ali, Ayman Ahmed, Hanaa A. M. Siam, Deena M. Abdelgadir, Olabanji A. Surakat, Olatunwa J. Olamiju, Daniel A. Boakye, Nabil Aziz, Kamal Hashim

**Affiliations:** 1 Onchocerciasis Control/Elimination Programme, National Programme for Prevention of Blindness (NPPB), Federal Ministry of Health, Khartoum, Sudan; 2 Department of Parasitology and Medical Entomology, Faculty of Medical Laboratory Sciences, University of Khartoum, Khartoum, Sudan; 3 Department of Immunology and Biotechnology, Tropical Medicine Research Institute, National Center for Research, Khartoum, Sudan; 4 Department of Medical Entomology, Blue Nile National Institute for Communicable Diseases, University of Gezira, Gezira, Sudan; 5 Department of Medical Entomology, National Public Health Laboratory, Federal Ministry of Health, Khartoum, Sudan; 6 Department of Epidemiology, Federal Ministry of Health, Khartoum, Sudan; 7 Department of Pure and Applied Zoology, Federal University of Agriculture, Abeokuta, Nigeria; 8 Department of Research and Development, Mission to Save the Helpless (MITOSATH), Jos, Nigeria; 9 Parasitology Department, Noguchi Memorial Institute for Medical Research, University of Ghana, Accra, Ghana; 10 The Carter Center, Khartoum, Sudan; 11 National Programme for Prevention of Blindness (NPPB), Federal Ministry of Health, Khartoum, Sudan

**Keywords:** Upper Atbara dam, Setit dam, Dam complex, *Simulium damnosum s.l.*, Distribution, Cross-border, Galabat sub-focus, Eastern Sudan

## Abstract

**Background:**

Onchocerciasis is caused by a nematode worm *Onchocerca volvulus*, which is transmitted in Sudan by black fly vectors of the *Simulium damnosum sensu lato* species complex. In Sudan, the disease is found in four foci where fast flowing rivers provide suitable breeding sites for the *Simulium* vector flies. The construction of dams and irrigation schemes for agricultural purposes has affected black fly breeding and distribution, such as in Merowe Dam in Abu-Hamed focus, where the perennially flowing water downstream of the Dam created new vector breeding sites, thereby, changing the pattern of disease transmission and creating public health problems. Based on this situation, this study was carried out to measure the effect of the Upper Atbara and Setit Dam complex on the distribution of *Simulium damnosum s.l.* breeding sites and on disease elimination in the Galabat sub-focus in eastern Sudan.

**Methods:**

Aquatic stages of *Simulium* were collected between October and November 2009, prior to the construction of the dam complex, and again in 2013 and 2015 while the dam complex construction was ongoing.

**Results:**

A total of 40 breeding sites were identified at the beginning of the study. After the construction of the dam complex in 2015, seventeen previously mapped breeding sites were inaccessible as they had been flooded by the dam complex’s lake when reach its maximum size. Three species were obtained from different locations: *S. damnosum s.l.*, *S. griseicolle*, and *S. adersi*.

**Conclusions:**

This study has shown a link between the construction of the dam complex and a reduction in the breeding sites of black fly vectors. This reduction has limited the Galabat sub-focus to a small area at the upper Atbara River which become the end of the focus. To sustain the success achieved in onchocerciasis control in the Galabat sub-focus, disease control and its vector control should be strengthened in the area cross-boarding Sudan and Ethiopia.

**Electronic supplementary material:**

The online version of this article (10.1186/s12879-019-4113-1) contains supplementary material, which is available to authorized users.

## Background

The River Nile is the longest river in the world [1]. The White Nile contributes a small amount to the annual flow discharge of the River Nile, but flows constantly throughout the year. The Blue Nile and its tributaries contribute by high amount of annual flow but on a highly seasonal basis [1, 2]. Although the River Nile is the main source of water in Sudan, the River Atbara and its tributaries are of most importance as seasonal water streams, creating distinct environment for local communities in eastern states [1].

A recent construction began on the Upper Atbara and Setit Dam complex [1, 2]. This is a twin dam consisting of Rumela Dam on the Upper Atbara River and Burdana Dam on the Setit River in eastern Sudan. The dams are located about 20 km upstream from the junction of the Atbara and Setit rivers and about 80 km to the south of Khashm El-Girba Dam [1]. The project began in 2010 and was finished in beginning of 2015. The two connected dams have a joined reservoir. Their total length is 13 km [3].

Black flies constitute a serious public health problem as vectors of human onchocerciasis and as a biting nuisance in many rural parts of the world [4, 5]. They breed in rivers in a constant flow of fast-moving water where they attach to rocks and plants and filter out suspended particles [5, 6]. Some human structures such as concrete dams and concrete-lined stream channels provide excellent developmental sites for larvae and pupae of black fly species. This may result in adverse environmental and health impacts, which may extend to other countries, as in the case of the River Nile [5, 7, 8].

In Sudan, human onchocerciasis is currently known in four main foci: Abu-Hamed in northern Sudan, Galabat in eastern Sudan, Radom in southern Darfur in the southwest, and Khor Yabus in Blue Nile region of the southeast [5]. Few studies have addressed the impact of dams on transmission dynamics of onchocerciasis in African countries [4, 9–12]. It has been suggested that onchocerciasis is the major environmental health problem around other dams in Africa [5, 9, 13]. In Sudan, few studies have highlighted the impact of dams on transmission dynamics of onchocerciasis. Biting activity of black flies was reported near Meridi and Roseires Dams, indicating that dam construction may affect the dynamics and extent of onchocerciasis transmission [14, 15]. Additionally, larvae of *S. damnsosum s.l.* were found in the spillways of Khashm El-Girba Dam after its construction [16]. Currently, Merowe Dam in Abu-Hamed focus has effectively shrunken the focus to a little over half of its original size and eliminated black fly breeding sites west to the town of Abu-Hamed [5].

The Galabat sub-focus is located along the banks of Atbara River and extends to the Ethiopian land in the Metema sub-focus [17, 18]. It has been recommended that any vector control activities based in this area would be operated by the Sudanese and Ethiopian governments [17]. Galabat started full continuous annual community- directed treatment with ivermectin (CDTI) in 2007. The prevalence of microfilariae was determined in a previous survey as 63.4% associated with a remarkable degree of pruritus and onchodermatitis [19]. The corresponding black fly infectivity rate in Galabat was 6.9/10,000 flies (95% CI = 1.1–16.4) [18]. The man-biting activity of *S. damnosum* increased in September–December, then decreased in February–June. The highest vector density and monthly biting rates (MBR) were recorded in September 2010 (*N* = 1163 flies, MBR = 6978 bites/person/month), whereas none were recorded from April to June. The hourly-based distribution of black flies showed a bimodal pattern with two peaks (07:00–10:00) and (14:00–18:00) in the Galabat sub-focus [20].

This study was conducted to document the breeding sites of onchocerciasis vector *S. damnosum s.l.* along Atbara River in the Galabat sub-focus, and to investigate the effect of the construction of the dam complex on the size of the sub-focus and the distribution of black fly vectors.

## Methods

### Study area

**Fig. 1:**
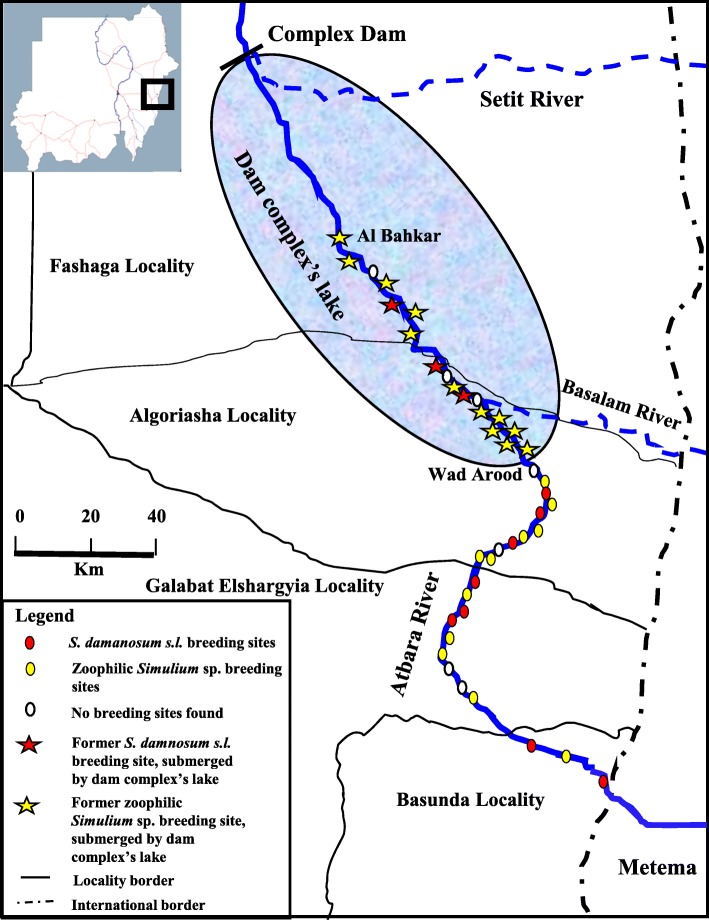
Map of the Galabat sub-focus showing the study sites before and after the construction of the Upper Atbara and Setit Dam complex

The Galabat sub-focus is found in eastern Sudan around the Atbara River (N 14° 06`–12° 57`, E 35° 56`–36° 09`) at an altitude range of 540–1040 m (Fig. 1). The water level at Atbara River increases generally in July to reach its peak in the rainy season from August to October, then decreases rapidly and forms isolated pools from March to May. The coordinates of each site from the first village in the sub-focus “Balashora” at the border with Ethiopia to the last village in the sub-focus “Al Bahkar” had been determined and listed in Additional file 1.

### Aquatic stages survey

During 2009, 2013, and 2015, investigation of 40 *Simulium* breeding sites were conducted from October and November of each year during the study period (Additional file 1). After its construction in 2015, the area in front of the dam complex was also surveyed for the presence of the vectors.

Aquatic stages (eggs, larvae, and pupae) of black flies were collected from different submerged plants like *Digitaria ciliaris*, *Kanahia laniflora* and *Cynodon dactylon* which are locally known as Dees, Guweer, and Nageela respectively [21]. The collected larvae and pupae were preserved in 80% ethyl alcohol for morphological identification following the taxonomic keys of Freeman and de Meillon (1953), Crosskey (1962), Davies and Crosskey (1991) [22–24].

## Results

Aquatic stages of *Simulium* black flies were collected from 40 breeding sites in the Galabat sub-focus. Details of locations and abundance of breeding sites are described in Additional file 1. Collected samples revealed three species: *S. damnosum s.l.* and two zoophilic species (*S. griseicolle* and *S. adersi*). Immature stages of the zoophilic species were predominantly encountered downstream of Atbara River, whereas, the *S. damnosum s.l.* was collected more from upstream of Atbara River where the fast current of water with many rapids was clearly notable.

Before the construction of the dam complex, the first breeding site for onchocerciasis vectors was found in Balashora site near the Ethiopian border [APOC and NPPB: Annual report of onchocerciasis in Galabat sub-focus/unpublished], and the last breeding site was in Al Bahkar site in north of the focus (Fig. 1). After the construction of the dam complex in 2015, 17 breeding sites located from the dam complex up to Um Gazaz site were totally flooded by water of the dam complex’s lake, covering an area of more than 100 km^2^ (Fig. 1). The 17 flooded sites were previously known as breeding sites of onchocerciasis vectors (Additional file 1). Therefore, the first breeding site was found near Wad Arood site (Fig. 1).

## Discussion

Construction of dams has adverse effects on the physical and biological environment [5, 9, 25]. In this study, aquatic stages of *Simulium* black flies were collected from 40 breeding sites during 2009 and 2013. Clearly, the construction of the Upper Atbara and Setit complex dam in 2015 has reduced the breeding sites of the vectors, as 17 previous breeding sites were covered by flooded water from the dam complex’s lake. This resulted in shrinking of the Galabat sub-focus size.

The findings of this study were consistent with previous studies conducted in Ghana [9] and Sudan [5], in which the breeding sites of black flies were reduced due to construction of dams. The chance of disease elimination in the Galabat sub-focus increased when the upper north limit of the sub-focus became a lake.

The identified aquatic stages were revealed to be anthropophilic species (*S. damnosum s.l.*) and two zoophilic species (*S. griseicolle,* and *S. adersi*). They shared same breeding sites, comprised of rocks and vegetation in the Atbara River, but they differed in abundance and distribution alongside the river. Most of *S. damnosum s.l.* were found upstream of the dam complex (towards the Ethiopian border) where the speed of water was very high and many rapids were found. *S. griseicolle* and *S. adersi* predominated downstream of the dam complex where the water current was low. This observation was mentioned early in previous studies [17, 26, 27].

The presence of high numbers of aquatic stages of *S. damnosum s.l.* at Balashora site, adjacent to the Ethiopian border, may support the idea of considering the Galabat sub-focus in Sudan and the Metema sub-focus in Ethiopia as one transmission zone. This consideration was supported previously by Raybould and White [26] who suggested control measures to be taken by the governments of Sudan and Ethiopia.

The nearest Sudanese focus to the Galabat sub-focus is more than 600 km at “Khor Yabus,” and the chance for new infection or recrudescence will be low if the disease is eliminated from Galabat. This is similar to the isolation of the Abu-Hamed focus after Merowe Dam was built in 2009 [5]. Conscientious monitoring of breeding sites in front of the dam complex is imperative. An agreement between Sudan and Ethiopia is required to eliminate the disease from the one transmission zone in the area.

## Conclusions

Construction of the Upper Atbara and Setit Dam complex and its artificial lake had changed the physical, biological, and socio-economic environment of the affected breeding sites in the Galabat sub-focus. The dam complex has produced a positive effect by reducing the breeding sites of black flies in the area. Agreement on an action plan for an effective disease control/elimination program must be achieved between Sudan and Ethiopia.

## Additional file

Additional file 1: Abundance of aquatic stages of *Simulium* black fly collected from different sites in Galabat sub-focus, during 2009, 2013 and 2015. (DOCX 26 kb)

## Data Availability

All data generated or analyzed during this study are included in this published article.

## Abbreviations

CDTI: Community-directed treatment with ivermectin
CI: Confidence interval
MBR: Monthly biting rate
*S*: *Simulium*
*s.l*: *sensu lato*
