# Supplementary material for: Notes on distribution of Simulium damnosum s. l. along Atbara River in Galabat sub-focus, eastern Sudan
Source: BMC Infect Dis. 2019 May 28;19:477. doi: 10.1186/s12879-019-4113-1 (PMC6540567; doi:10.1186/s12879-019-4113-1)
Supplement: Supplementary file 1 — Abundance of aquatic stages of Simulium black fly collected from different sites in Galabat sub-focus, during 2009, 2013 and 2015. (DOCX 26 kb) [file 12879_2019_4113_MOESM1_ESM.docx]

**Additional file 1. Abundance of aquatic stages of *Simulium* black fly collected from different sites in Galabat sub-focus, during 2009, 2013 and 2015**

| **Village** | **Coordinates** | **Locality** | **2009** | | | **2013** | | | **2015** | | |
| --- | --- | --- | --- | --- | --- | --- | --- | --- | --- | --- | --- |
|  |  |  | **Eggs** | **Larvae** | **Pupae** | **Eggs** | **Larvae** | **Pupae** | **Eggs** | **Larvae** | **Pupae** |
| Balashora | N: 13° 02.148´  E: 036° 09.666´ | Basunda | - | +++ | ++ | + | +++ | ++ | + | ++ | ++ |
| Kunaina Tabaldyia | N: 13° 09.141´  E: 036° 03.630´ | Basunda | - | ++ | + | - | - | - | ++ | ++ | ++ |
| Um Sai | N: 13° 11.851´  E: 036° 02.657´ | Basunda | - | + | + | + | + | + | + | + | + |
| Hilat Khatir | N: 13° 12.911´  E: 036° 01.969´ | Galabat Elshargyia | ++ | +++ | +++ | - | +++ | - | + | +++ | +++ |
| Raddah | N: 13° 15.583´  E: 036° 01.782´ | Galabat Elshargyia | - | + | + | - | - | ++ | - | ++ | - |
| Sundus | N: 13° 18.166´  E: 036° 01.375´ | Galabat Elshargyia | - | + | + | - | - | - | - | - | - |
| Azazza | N: 13° 19.741´  E: 036° 02.149´ | Galabat Elshargyia | - | + | + | + | + | + | + | + | + |
| Abd Elwahab | N: 13° 20.004´  E: 036° 02.091´ | Galabat Elshargyia | + | + | + | + | ++ | ++ | + | ++ | ++ |
| Um Khutur | N: 13° 21.378´  E: 036° 03.773´ | Galabat Elshargyia | + | ++ | ++ | - | ++ | ++ | + | ++ | ++ |
| Um Addara | N: 13° 22.888´  E: 036° 04.089´ | Galabat Elshargyia | - | ++ | - | - | ++ | - | + | ++ | ++ |
| Um Deesa | N: 13° 24.611´  E: 036° 05.178´ | Galabat Elshargyia | + | ++ | ++ | + | + | + | + | +++ | ++ |
| Jumaiza | N: 13° 25.343´  E: 036° 05.457´ | Galabat Elshargyia | + | +++ | +++ | - | +++ | ++ | + | +++ | ++ |
| Birkat Nurain | N: 13° 28.764´  E: 036° 09.585´ | Algoriasha | - | ++ | + | + | + | + | + | + | + |
| Mushra’a Fursan | N: 13° 29.798´  E: 036° 10.882´ | Algoriasha | - | ++ | + | - | - | - | + | + | + |
| Lafat Gunoon | N: 13° 29.844´  E: 036° 12.805´ | Algoriasha | - | - | - | - | - | - | - | - | - |
| Um Tawakul | N: 13° 30.725 ´  E: 036°14.276 ´ | Algoriasha | + | ++ | + | - | ++ | ++ | + | ++ | ++ |
| Marbata | N: 13° 31.137 ´  E: 036° 15.637 ´ | Algoriasha | - | + | + | - | ++ | ++ | + | ++ | ++ |
| Wad Koli | N: 13° 32.662 ´  E: 036° 15.235 ´ | Algoriasha | + | ++ | + | - | - | - | + | ++ | ++ |
| Mudeiria | N: 13° 35.107 ´  E: 036°18.825 ´ | Algoriasha | - | - | - | - | ++ | ++ | + | +++ | +++ |
| Tibar | N: 13° 37.050´  E: 036° 17.800´ | Algoriasha | - | ++ | + | - | ++ | ++ | - | + | + |
| Allali | N: 13° 37.716´  E: 036° 18.676´ | Algoriasha | - | +++ | ++ | - | +++ | +++ | - | +++ | +++ |
| Wad Arood | N: 13° 40.194´  E: 036° 16.549´ | Algoriasha | - | + | + | - | - | - | - | - | - |
| Eldabi Island | N: 13° 41.277´  E: 036° 15.258´ | Algoriasha | - | - | - | - | - | - | - | + | + |
| Um Gazaz (*) | N: 13° 42.980´  E: 036° 14.001´ | Algoriasha | - | - | - | - | - | - | - | - | - |
| Aldabkar (*) | N: 13° 45.684´  E: 036° 12.235´ | Algoriasha | - | ++ | + | - | - | - | - | - | - |
| Sharbob (*) | N: 13° 47.564´  E: 036° 10.249´ | Algoriasha | - | - | - | - | - | - | - | - | - |
| Hilat Barno (*) | N: 13° 49.673´  E: 036° 08.137´ | Algoriasha | - | + | - | ++ | +++ | +++ | - | - | - |
| Korani (*) | N: 13° 50.573´  E: 036° 07.356´ | Algoriasha | + | ++ | ++ | ++ | ++ | ++ | - | - | - |
| Safawa (*) | N: 13° 51.502´  E: 036° 07.058´ | Algoriasha | - | ++ | + | - | - | - | - | - | - |
| Deggi-Mogran (*) | N: 13° 52.675´  E: 036° 06.056´ | Algoriasha | - | +++ | ++ | - | +++ | +++ | - | - | - |
| Kona Zabarma (*) | N: 13° 53.358´  E: 036° 05.475´ | Algoriasha | - | - | - | - | - | - | - | - | - |
| Al Dahaab (*) | N: 13° 55.499´  E: 036° 04.059´ | Algoriasha | - | + | + | - | + | + | - | - | - |
| El Draabi (*) | N: 13° 57.442´  E: 036° 04.236´ | Algoriasha | - | + | + | - | ++ | ++ | - | - | - |
| Wad Gabir (*) | N: 14° 00.553´  E: 036° 01.253´ | Fashaga | - | - | + | - | - | - | - | - | - |
| Hilaiba (*) | N: 14° 00.794´  E: 036° 01.691´ | Fashaga | - | + | + | - | - | - | - | - | - |
| Ajab Seido (*) | N: 14° 02.924´  E: 036° 00.688´ | Fashaga | + | +++ | ++ | +++ | +++ | +++ | - | - | - |
| Mantik (*) | N: 14° 04.7425´  E: 035° 57.387´ | Fashaga | - | - | - | - | - | - | - | - | - |
| Hilat Nouh (*) | N: 14° 05.085´  E: 035° 58.143´ | Fashaga | - | - | - | - | - | - | - | - | - |
| Shashola (*) | N: 14° 05.497´  E: 035° 57.076´ | Fashaga | - | + | - | - | - | - | - | - | - |
| Al Bahkar (*) | N: 14° 06.577´  E: 035° 56.436´ | Fashaga | + | ++ | ++ | - | - | - | - | - | - |

(*) Previous breeding site of black fly

(+) rare; (++) few; (+++) abundant
